# Effect of Film Thickness on the Self-Assembly of CBABC Symmetric Pentablock Terpolymer Melts under 1D Confinement: A Dissipative Particle Dynamic Study

**DOI:** 10.3390/ma16216862

**Published:** 2023-10-25

**Authors:** Yingying Guo

**Affiliations:** School of Science, Qingdao University of Technology, Qingdao 266525, China; gyyhappy@outlook.com

**Keywords:** symmetric pentablock terpolymres, phase behavior, DPD simulation

## Abstract

The study investigates the impact of film thickness on the phase behavior of pentablock terpolymers, denoted as *C*_3_*B*_3_*A*_6_*B*_3_*C*_3_, when subjected to wall confinement by utilizing the dissipative particle dynamics method. Phase diagrams were constructed to elucidate how factors such as block–block interaction strength, film thickness, and wall properties affect the self-assembly structures. In cases where the wall exhibits no preference for any of the blocks, lamellae phases with orientations perpendicular to the wall are observed. The order–disorder transition (ODT) temperature is found to be influenced by the interaction between the polymer and the wall in thin confinement scenarios. When the wall displays a preference for specific blocks, the orientation of lamellae structures undergoes variations. Lamellae tend to align parallel to the wall when the wall favors A or C blocks, and they orient perpendicularly when B blocks are favored. Furthermore, the mechanical properties of the lamellae structures are related to the conformations of the polymer chains. Structures where chains predominantly adopt a loop conformation exhibit enhanced elastic properties. The ratio of looping to bridging conformations can be adjusted by altering the film thickness and wall selectivity.

## 1. Introduction

The use of block copolymers (BCPs) to tailor self-assembled nanostructures with specific nanodomains at particular length scales has attracted considerable attention across various fields, such as lithography, drug delivery systems, nanocomposites, and nanodevices [1,2]. Multiblock copolymers, a subset of BCPs, are particularly noteworthy due to their capacity to create innovative self-assembled structures that can enhance information storage capabilities. This is achieved by manipulating the number, sequence, and functionality of the constituent blocks. Consequently, these advanced multiblock copolymers are expected to be the next generation of materials [3,4,5].

Linear pentablock terpolymers, denoted as ABCBA, comprising five distinct blocks, represent a fundamental model for investigating the self-assembly of multiblock copolymers [6]. Both experimental studies [7,8,9,10,11] and computational simulations [12,13] have been employed to explore the phase behavior of these terpolymers in bulk. Various structures have been identified, including the lamellar phase structure (LAM3), the double gyroid (Q230) network structure, and the hexagonal perforated lamellae (HPL) structure. These structures have been observed in linear pentablock terpolymers, specifically poly(tert-butyl acrylate)-b-polystyrene-b-poly(ethylene oxide)-b-polystyrene-b-poly(tert-butyl acrylate) (PtBA-PS-PEO-PS-PtBA, TSOST), with varying lengths of the PEO block in the middle [7]. Shi et al. [12] conducted an extensive examination of the phase behavior of linear ABCBA pentablock terpolymers using 3D self-consistent field theory, making comparisons with linear ABC triblock terpolymers. Their research revealed that when the interaction parameters among the blocks are equal, pentablock terpolymers exhibit diverse complex network structures and binary crystalline phases. In addition to studying the phase behavior, Bates et al. carried out a comparative analysis of the tensile properties between non-frustrated poly(ethylene oxide)-b-polystyrene-b-polyisoprene-b-polystyrene-b-poly(ethylene oxide) (OSISO) pentablock terpolymers and OSI triblock terpolymers with an O70 microphase structure [10]. Their conclusion was that OSISO pentablock terpolymers exhibit superior tensile properties compared to OSI triblock terpolymers. This superiority in tensile properties is attributed to the unique topological structures inherent to ABCBA, enabling polymer chains to adopt both loop and linear conformations. Given their enhanced mechanical properties and the ease with which they form continuous microphase structures, pentablock copolymers offer significant potential for applications when precisely tailored [12].

Compared to bulk self-assembly, thin-film self-assembly exhibits unique properties due to the influence of surfaces and interfaces. When subjected to spatial confinement, thin films are not solely affected by intrinsic factors such as block–block immiscibility, block compositions, and chain architectures; they are also influenced by extrinsic factors. These extrinsic factors encompass the degree of confinement and interactions between polymers and surfaces [14], which play a crucial role in determining the properties and behavior of self-assembled thin films. Since the stretching or compression of polymer chains induced by confinement is energetically unfavorable, block copolymers tend to self-assemble into morphologies that relieve the stretching/compression or mitigate the entropic penalty with favorable enthalpic interactions at the confined substrate or free surface [15]. Furthermore, surface properties are important parameters for controlling the self-assembly of block copolymer thin films. They have a profound impact on both the stability of the microstructure [16] and its orientation [17,18]. Consequently, block copolymers under confinement are widely employed to achieve highly ordered nanopatterns or to control the orientation of these nanopatterns [19].

The simplest scenario involving block copolymers (BCPs) under confinement is the one-dimensional (1D) confined system, where block copolymers are placed on a substrate with the other surface exposed to the air [1]. This situation can be theoretically represented by confining the polymer between two impenetrable surfaces, with assigned surface potentials based on their surface properties [20]. Such 1D confinement serves as a fundamental basis for predicting morphologies under 2D or 3D confinements [21]. Numerous experimental [19,21,22,23,24,25,26] and theoretical studies [20,27,28,29,30] have been conducted to investigate the behavior of BCPs under 1D confinement. Researchers have investigated a wide range of confined block copolymer morphologies by adjusting parameters like wall separation distance, polymer chain length, and surface properties. These structures include commonly observed ones such as lamellae, cylinders, spheres, and gyroid structures. Different polymer architectures have been thoroughly examined, including linear AB diblock, ABC triblock, multiblock A(BC)_n_, star A_2_B, bottlebrush, and H-shape copolymers. However, studies specifically focusing on symmetric pentablock copolymers under confinement are scarce, to the best of the author’s knowledge.

In this study, we employed computer simulations to investigate the behavior of symmetric CBABC pentablock terpolymers under wall confinement. Computer simulations offer a cost-effective and time-efficient alternative to experiments, allowing for the convenient modification of polymer chain parameters and environmental variables. We have chosen the dissipative particle dynamic method (DPD) as our simulation approach, given its successful application in studies of polymer systems [30,31] under confinement. The paper will address essential factors concerning the impact of confinement, specifically focusing on wall selectivity and the confined thickness.

## 2. Simulation Methods

**Figure 1:**
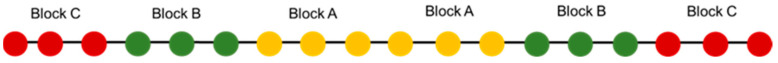
Schematic showing the structure of a linear symmetric $C_{3}B_{3}A_{6}B_{3}C_{3}$ pentablock terpolymer. (Please note that C, B, and A blocks are consistently represented as red, green, and yellow throughout the article).

The dissipative particle dynamics (DPD) method was employed to investigate the self-assembly behavior of symmetric $C_{3}B_{3}A_{6}B_{3}C_{3}$ pentablock terpolymers, as shown in Figure 1, under wall confinement. In the simulations, all polymers were coarse-grained and composed of DPD beads of a uniform size.

The motion of each DPD bead is governed by Newton’s second law. The total force acting on each bead, denoted as ${\vec{f}}_{i\;}$, comprises four components. Among these, three components—the conservative force ${\vec{F}}_{ij}^{C}$, the dissipative force ${\vec{F}}_{ij}^{D}$, and the random force ${\vec{F}}_{ij}^{R}$ [32]—become effective when the distance between beads i  and j falls within the cut-off radius $r_{c}$. The conservative force, ${\vec{F}}_{ij}^{C}$, is a soft-repulsive force and is given by

(1) $${\vec{F}}_{ij}^{C}=\left\{\begin{array}{c} a_{ij}\left(1-r_{ij}/r_{c}\right){\hat{r}}_{ij}\left(r_{ij}<r_{c}\right)\\ 0\;\left(r_{ij}\geq r_{c}\right) \end{array}\right.$$

where ${\vec{r}}_{ij}={\vec{r}}_{i}-{\vec{r}}_{j}$, $r_{ij}=\left|{\vec{r}}_{ij}\right|$, ${\hat{r}}_{ij}={\vec{r}}_{ij}/\left|{\vec{r}}_{ij}\right|$. $a_{ij}$ is the maximum repulsion between beads i and j, and $r_{c}$ is the cut-off radius with value 1.0. 

In our model systems, CBABC pentablock terpolymers, consisting of A, B, and C beads, were subjected to wall (W bead) confinement. To simplify the model, we assumed that the interaction parameters among the polymer beads were equal, denoted as $a_{AB}=a_{BC}=a_{AC}$. The study examined the influence of wall selectivity on polymers under different levels of polymer–polymer segregation strength (40 or 80). The interaction parameters among the beads are detailed in Table 1. Additionally, a fourth force component, the spring force ${\vec{F}}_{ij}^{S}$, was introduced. This force acts between beads connected by covalent bonds to mimic polymer chains. The spring force ${\vec{F}}_{ij}^{S}$ follows a simple harmonic potential with a spring constant *k* = 8.0.

All simulations were conducted within the NVT ensemble, beginning with a homogeneous melt of pentablock terpolymer $C_{3}B_{3}A_{6}B_{3}C_{3}$ in a periodic box with the dimensions $60.0r_{c}\times 60.0r_{c}\times hr_{c}$, where *h* represents the film thickness. The study involved altering the h value to investigate the effect of film thickness on self-assembly morphologies. Specifically, cases where h=3, 4, 5, 6, 9, 11, 21,  and 41 were examined. For wall confinement, two identical rigid walls composed of *W* beads were positioned at the upper and lower *z* boundaries of the periodic box. These walls were built with a face-centered cubic structure, facing the (100) plane toward the melt. The lattice spacing was 0.855 $r_{c}$. The computations were allowed to equilibrate for a minimum of 500,000 timesteps, with a consistent timestep value of Δt=0.04 employed for all simulations.

## 3. Results and Discussion

In the self-assembly of polymers under wall confinement, film thickness and wall selectivity play crucial roles in determining the equilibrium aggregate structures. They are as significant as factors like chain architecture, block length, and the natural chemical differences between the blocks. This study investigated the impact of confinement on the morphological behavior of symmetric CBABC pentablock terpolymer melts. The observed phase behavior arose from the intricate interplay among polymer–polymer segregation strength, confined film thickness, and substrate selectivity. Notably, in our study, the composition ratio among all blocks was 1:1:1, aligning with the lamellar structure observed in bulk self-assembly [12].

### 3.1. Nonselective Wall

In this section, we examine the phase behavior of polymers in the presence of a nonselective wall, where the wall exhibits no preference for any of the blocks ($a_{AW}=a_{BW}=a_{CW}$). We specifically studied the effect of film thickness on the self-assembly behavior under two distinct scenarios: (1) when the wall strongly attracts all blocks ($a_{polymer-wall}=25$); (2) when the wall strongly repels all blocks $\left(a_{polymer-wall}=120\right)$.

**Figure 2:**
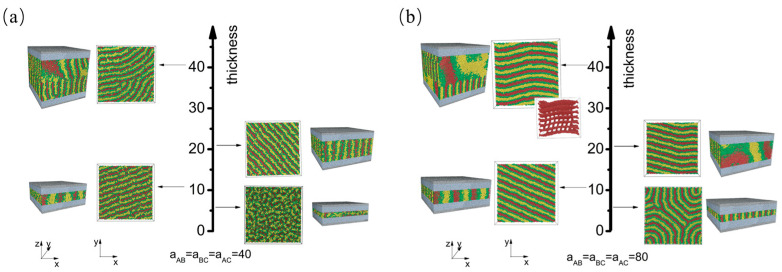
Thickness-dependent self-assembly morphology of CBABC pentablock terpolymers at (**a**) a weak block–block interaction strength and (**b**) a strong block–block interaction strength when the polymer–wall interaction energy is weak ($a_{polymer-wall}=25$). The corresponding film thicknesses are h=6, h=11, h=21, and h=41.

We began with the first scenario, where the wall exhibited a strong attraction to all blocks. Our aim was to gain a thorough understanding of how film thickness influences self-assembly morphologies, while taking into account varying levels of polymer–polymer interaction strength. To facilitate this, we constructed a phase diagram (Figure 2) under two different polymer–polymer interaction strengths: $a_{AB}=a_{BC}=a_{AC}=40$, representing polymers in a weak segregation regime; and $a_{AB}=a_{BC}=a_{AC}=80$, representing polymers in a strong segregation regime.

When the interaction strength among blocks is weak ($a_{AB}=a_{BC}=a_{AC}=40$), a disordered phase is observed for a thin film with a thickness of h=6 (Figure 2a). As the film thickness increases, specifically, when h≥11, the chains have more opportunities to interact. To minimize unfavorable interactions between distinct blocks, the chains start to phase-separate. Perpendicular lamellae structures are observed at h=11 and 21. In these structures, lamellae are oriented perpendicular to the wall, with each layer aligned parallel to the others. When the thickness increases further to h=41, the polymer chains self-assemble into a fingerprint lamellae structure.

As the block immiscibility increases ($a_{AB}=a_{BC}=a_{AC}=80$) (Figure 2b), phase separation becomes more pronounced, leading to the formation of fingerprint lamellae, even in thin films for which h=6. As the film thickness increases to 21, similar to the behavior observed in the weak segregation regime, the polymer chains self-assemble into perpendicular lamellae structures, with each layer aligned parallel to the adjacent layers. However, as the thickness further increases to h=41, a deviation from the weak segregation regime becomes apparent. Notably, a cross lamellae morphology characterized by a 90-degree twist angle is observed. A closer examination of this distinct arrangement reveals a Scherk surface pattern. This pattern bears a remarkable resemblance to the cross-cylinder structure that arises from the self-assembly of cylinder-forming asymmetric diblock copolymers confined between two parallel flat surfaces [33].

**Figure 3:**
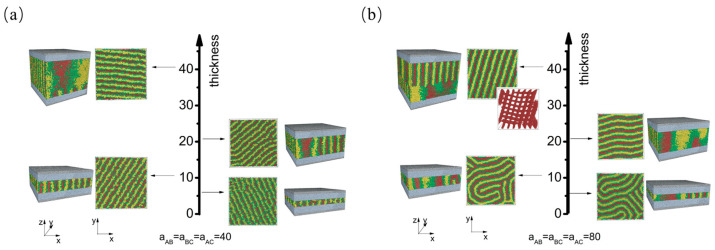
Thickness-dependent self-assembly morphology of CBABC pentablock terpolymers at (**a**) a weak block–block interaction strength and (**b**) a strong block–block interaction strength when the polymer–wall interaction energy is weak ($a_{polymer-wall}=120$). The corresponding film thicknesses are h=6, h=11, h=21, and h=41.

Next, we explored the scenario where the wall exhibits strong repulsion $\left(a_{polymer-wall}=120\right)$ toward all blocks (Figure 3). When the interaction strength among polymer blocks is weak, perpendicular lamellae structures with parallel monolayers are dominant (Figure 3a). Compared with morphologies obtained at weak polymer–wall interactions (Figure 2a), we observe that a substantial repulsive interaction between the polymer and the wall prompts the polymer melt to adopt an ordered lamellae structure, even at a minimal film thickness (h=6). It is important to note that the order–disorder transition (ODT) temperature represents the temperature at which a material undergoes a phase transition from an ordered or structured state to a disordered or random state, or vice versa. Considering that the interaction energy is inversely proportional to temperature, the results reveal that increasing the polymer–wall interaction leads to an upward shift of the order–disorder transition (ODT) temperature. This observation aligns with the results presented by Yong et al. [34]. When the interaction strength among polymers reaches 80, various structures emerge depending on the film thickness (as shown in Figure 3b). These structures include fingerprint lamellae obtained at a film thickness of h≤11. As the film thickness increases to 21, perpendicular lamellae with parallel monolayers emerge. Further increasing the thickness to 41 results in the formation of a cross lamellae structure. Compared with the structures obtained at different polymer segregation strengths, we observe that lamellae structures with parallel monolayers are more likely to form with weak block–block interactions.

**Figure 4:**
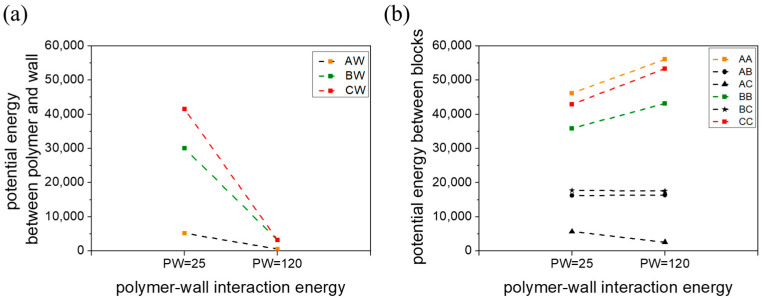
(**a**) Block–block potential energy and (**b**) block–wall potential energy concerning polymer–wall interaction energy at a film thickness of h=6.

The above demonstration highlights that increasing the interaction parameter between the polymer and the wall can have significant effects on the phase behavior of polymers, particularly in thin films under a weak segregation regime. To better understand this phenomenon, we calculated the potential energy (Figure 4) associated with interactions between different blocks ($E_{AA},\;E_{BB},\;E_{CC},\;E_{AB},\;E_{AC},\;E_{BC}$) and interactions between the block and the wall ($E_{AW},\;E_{BW},\;E_{CW}$). 

As depicted in Figure 4a, the potential energies between the blocks and the wall are notably lower when there is a high polymer–wall interaction ($a_{polymer-wall}=120$) compared to a low polymer–wall interaction ($a_{polymer-wall}=25$). This phenomenon can be ascribed to the strong repulsion experienced by the polymer beads toward the wall. To alleviate this unfavorable interaction with the wall, polymer beads tend to aggregate together. Given that the interaction parameter ($a_{ij}=40,\;i=A,\;B,\;C;i\neq j$) between different types of beads is larger than that of beads of the same type ($a_{ij}=25,\;i=A,\;B,\;C;i=j$), the latter tend to aggregate. This tendency promotes the phase separation of individual blocks, resulting in a significantly increased interaction energy among beads of the same type when there is high polymer–wall interaction, as shown in Figure 4b. Furthermore, we observe a decrease in $E_{AC}$ under conditions of high polymer–wall interaction strength. This reduction occurs because beads of the same type aggregate to minimize interaction enthalpy, leading to stretched chains. This stretching, in turn, facilitates the formation of ordered structures.

In summary, the phase diagram, concerning the film thickness and polymer segregation strength, indicates that the preferred configuration includes lamellae with a perpendicular orientation to the wall. This phase behavior closely resembles what is typically observed in bulk systems. This observation can be attributed to the fact that, with nonselective walls, the adsorption energy between the wall and the block copolymer does not play a crucial role. The perpendicular orientation of the lamellae allows the film to retain the lamellar spacing that is typically favored in bulk conditions [31]. Furthermore, our results regarding the influence of polymer–wall interaction under weak polymer segregation reveal that the order–disorder transition (ODT) temperature is significantly affected by interfacial interactions. The potential energy calculations offer a quantitative insight into the energies governing this behavior.

### 3.2. Selective Wall

#### 3.2.1. A-Block- and C-Block-Selective Wall

**Figure 5:**
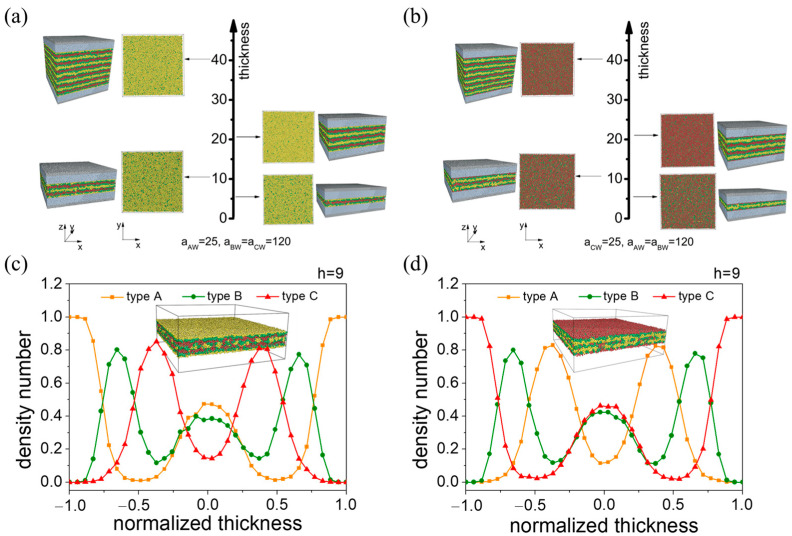
Thickness-dependent self-assembly morphology of CBABC pentablock terpolymers under selective confinement: (**a**) A-selective wall, $a_{AW}=25$; (**b**) C-selective wall, $a_{CW}=25$. Density profile and snapshot for self-assembly morphology under (**c**) an A-selective wall and (**d**) a C-selective wall at a film thickness of h=9. The normalized thickness is defined as $\frac{z-0.5*h}{0.5*h}$, where z is the distance to the upper wall, and h is the film thickness. The labels ‘type A’, ‘type B’, and ‘type C’ consistently refer to A beads, B beads, and C beads, respectively, throughout the article.

We began by examining confining walls with a strong affinity for the A component. In this case, parallel lamellae structures are consistently observed, regardless of film thickness. Unlike the perpendicular lamellae structures observed with nonselective walls, where the lamellae stand perpendicular to the wall, the lamellae in parallel structures lie parallel to the wall. The lamellae structure comprises v monolayers that vary with film thickness, as depicted in Figure 5. Here, v denotes the number of layers formed by the C block. In other words, the lamellar structure exhibits a periodicity of v. To gain a deeper understanding of the lamellae structures, we created number density profiles across the various film thicknesses. The density profiles, as illustrated in Figure S1(a1–a4), reveal a lamellar structure characterized by alternating peaks of A, B, and C blocks. The foremost layers, which appear at the polymer–wall interfaces, primarily consist of A blocks (as visualized in the xy plane in Figure 5a and Figure S1a). This arrangement arises from the wall’s preferential adsorption of A beads. As the film thickness decreases from *h* = 41 to 6, the periodicity of the structure diminishes. The value of the periodicity corresponds to *h* = 6, 3, 2, and 1, respectively, to match the film thickness. 

Similarly, when the wall shows a preference for the C blocks, parallel lamellar phases with ν monolayers are observed, as shown in Figure 5b and Figure S1b. However, in this context, ν signifies the number of layers formed by the A block. The variation in lamellar periodicity with film thickness is similar to that of the A-selective wall scenario. The key distinction lies in the composition of the layers adjacent to the wall and the polymer: they are now predominantly formed of C beads. This can be attributed to the wall’s preferential adsorption of C blocks.

Previous studies have reported that for AB diblock copolymers under confinement, the lamellae adopt a perpendicular alignment when the system experiences significant structural frustration between parallel lamellae with v and v+1 monolayers [31]. Given that at h=11, v=2 and at h=6, v=1, we chose a film thickness of h=9 to examine the self-assembly morphologies in between these values. Contrary to the expected perpendicular lamellae, we observed parallel lamellae with a middle layer composed of all blocks (as shown in Figure 5c,d).

**Figure 6:**
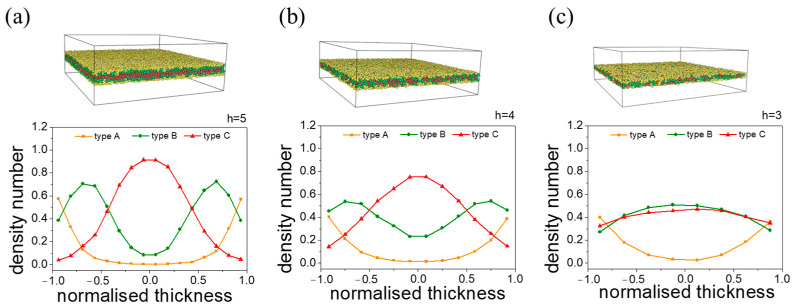
Self-assembly morphologies and the corresponding density number distribution along the *z*-axis at ultrathin film thicknesses under an A-selective wall: (**a**) h=5; (**b**) h=4; (**c**) h=3. The normalized thickness is defined as $\frac{z-0.5*h}{0.5*h}$, where z is the distance to the upper wall, and h is the film thickness.

When we further reduced the thickness from h=5 to 3 in unit steps for an A-selective wall, we observed that the polymer blocks gradually mixed. This is evidenced by the gradual disappearance of the two B peaks shown in Figure 6. The cause can be attributed to limited confinement in the z direction, where the chains lack sufficient space to expand. However, since the wall primarily selects for A beads, the majority of A beads are distributed near the wall rather than at the center. Comparing the density numbers obtained at h=6, where a structure with distinct A, B, C layers is shown (Figure S1a4), we observe that while two distinct B peaks can still be seen at h=5, the middle layer no longer consists solely of C blocks (Figure 6a). Therefore, a film thickness of h=6 can be considered the minimum requirement for a single periodic lamella. The phase behavior observed with a C-selective wall follows a similar trend; the corresponding morphology and density number profiles are available in Figure S2.

To understand the evolution process of parallel lamellae morphologies with periodicity (v), we plotted and compared snapshots at different timesteps for film thicknesses h=6 (Figure S3) and h=21 (Figure S4) in the presence of an A-selective wall. In all simulations, the chains start with a random distribution between two confinements (Figures S3(a1,a2) and S4(a1,a2)). Since the wall has an affinity for A blocks, the chains respond rapidly to the wall, resulting in a layer mainly composed of A beads observed at t=100 DPD timesteps (Figures S3(b1,b2) and S4(b1,b2)). For h=6, the morphology gradually evolves into a mixed lamellar structure, with the center layer mainly consisting of B beads and C beads (Figure S3(c1,c2)). As B blocks are directly connected to A blocks, the B beads gradually separate from the C beads as the A beads move to the wall (Figure S3(d1,d2)). Finally, lamellae with distinct A, B, and C nanodomains are observed (Figure S3(e1,e2)). In contrast, for h=21, we initially observe two lamellae structures with periodicity v=1 next to the top and bottom walls (Figure S4(c1,c2)). The part in between the two lamellae consists of a mixed lamellar structure formed of A, B, and C beads. Due to the lower interaction energy between beads of the same type, these common-type beads tend to aggregate together, leading to the observation of lamellae with a periodicity of v>1 (Figure S4(d1,d2,e1,e2)). From the analysis above, it becomes evident that wall selectivity guides the formation of parallel lamellae structures.

The microphase structure derived from the bulk assembly of symmetric ABCBA pentablock terpolymers has been reported to possess superior mechanical properties compared to similar morphologies formed by linear ABC terpolymers [10]. But how do these mechanical properties fare under confinement? To address this, we compared the tensile modulus of films derived from CBABC pentablock terpolymers with C/A selectivity to those from ABC triblock terpolymers with A selectivity. We conducted tensile tests on films at h=6, where the molecules self-assemble into a lamellar structure with a single period. For the tensile test, we subjected the film to uniaxial deformation along the z direction and calculated the stress $\tau_{zz}$. The strain $\varepsilon_{zz}$ is obtain by $\frac{L-L_{0}}{L_{0}}$, where the $L_{0}$ is the original box size in the z direction, and L is the box length as a function of time after deformation. The tensile modulus can then be obtained by $E=\frac{\sigma_{zz}}{\varepsilon_{zz}}$.

**Figure 7:**
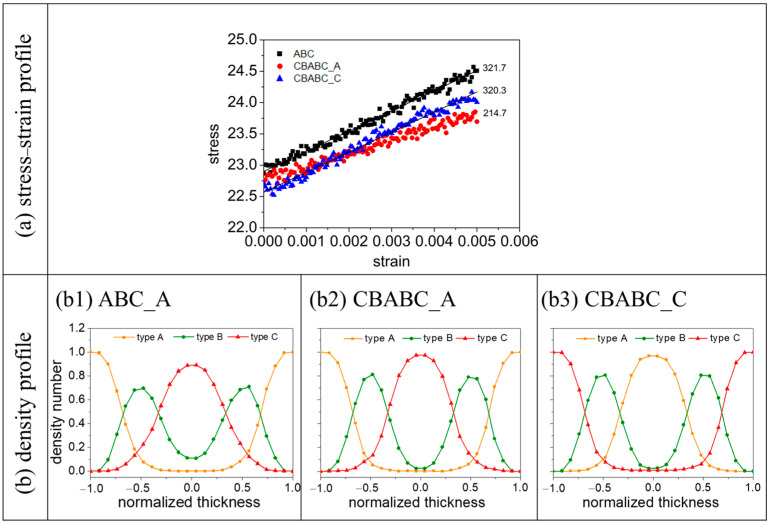
(**a**) Stress–strain curve and (**b**) number density profile for ABC_A, CBABC_C, and CBABC_A. The normalized thickness is defined as $\frac{z-0.5*h}{0.5*h}$, where *z* is the distance to the upper wall and h=6.

As shown in Figure 7a, for the CBABC pentablock terpolymers, the tensile modulus of the film derived from the C-selective wall (CBABC_C) is significantly higher than that from the A-selective wall (CBABC_A). To further investigate this phenomenon, we analyzed the chain conformation by calculating the mean square radius of gyration $R_{g}^{2}$ (Table 2) and the mean square end-to-end distance $R_{EE}^{2}$ (Table 3) of the chain. Upon comparing the $R_{g}^{2}$ for the two cases, we observe that chains in the CBABC_C scenario are more extended than those in the CBABC_A scenario. Given the chain architecture, where C blocks are positioned at both ends of the chain and A blocks are centrally located, a preference for C blocks by the wall allows both ends to simultaneously interact with the top and bottom walls. This results in the chain stretching in the z direction and forming a “bridge” between different nanodomains. In this case, the chain conformation is referred to as the bridging conformation (Figure S5a). On the contrary, if the wall has an affinity for A blocks, the central blocks of the chain will likely interact with the wall, resulting in a folded chain conformation with both end blocks situated on the same nanodomain at the center of the film. In this case, the chain conformation is referred to as the looping conformation (Figure S5b). This hypothesis is supported by the $R_{g,⊥}^{2}$, where the value for CBABC_C is nearly double that of CBABC_A, indicating that most chains under the C-selective wall extend in the z direction.

**Figure 8:**
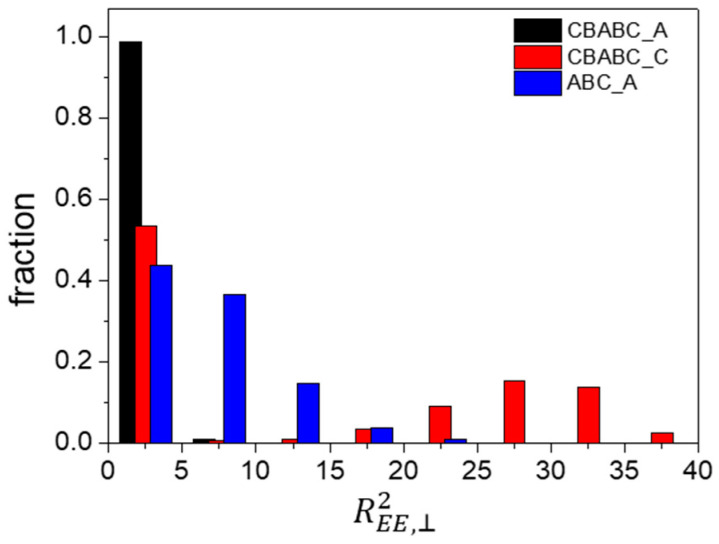
Histogram showing the perpendicular component $R_{EE,⊥}^{2}$ with various conditions at a film thickness of h=6.

**Figure 9:**
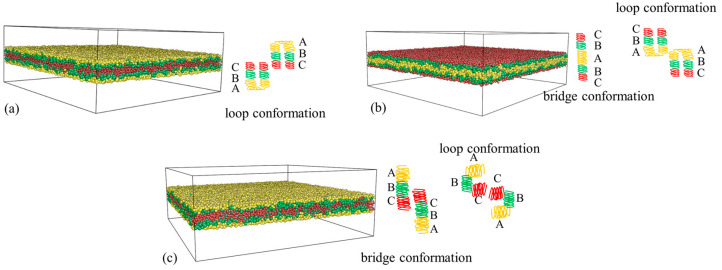
Typical chain conformation in the lamellae structure of (**a**) CBABC_A, (**b**) CBABC_C, and (**c**) ABC_A.

Further analysis of the distribution of the perpendicular component of $R_{EE}^{2}$ for various scenarios (Figure 8) revealed that 99% of the chains have an $R_{EE,⊥}^{2}$ value of less than five for an A-selective wall. Additionally, the average value of $R_{EE,⊥}^{2}$ for CBABC_A is approximately 0.73. Therefore, we infer that chains predominantly adopt a loop conformation (Figure 9a) under the influence of the A-selective wall. When the wall preference shifts to C blocks, approximately 46% of the chains have an $R_{EE,⊥}^{2}$ larger than 10 for the C-selective wall. Given that the maximum distance between two B peaks at h=6 is around 3$r_{C}$ (Figure 7(b2,b3)), it appears that both looping and bridging conformations (Figure 9b) coexist in the presence of the C-selective wall, with a roughly equal distribution between the two conformations.

We further compared the tensile modulus of the $A_{6}B_{6}C_{6}$ triblock terpolymers (ABC_A), where the A blocks are selective to the wall, with that of CBABC_C (Figure 7a). The tensile modulus for ABC_A is similar to that of CBABC_C. Upon examining the $R_{g}^{2}$, we observed that the chains in ABC_A exhibit a more folded conformation. This folding occurs because both the top and bottom walls have an affinity for A blocks, causing the chain to curl to minimize unfavorable interactions between the B/C blocks and the wall. 

Approximately 46% of the chains in ABC_A have an $R_{EE,⊥}^{2}$ value larger than five, as shown in Figure 8. However, the distance between adjacent layers formed by A and B beads is only about 2.2$r_{C}$ (Figure 7(b1)). Additionally, we observe that the value of $R_{EE,\;⊥}^{2}$ for CBABC_C is approximately twice that of ABC_A. These data indicate that in ABC_A, roughly half of the chains have A beads in contact with the wall, while the C beads are positioned in the central layer. Furthermore, the central layer in ABC_A comprises a blend of B and C blocks, in contrast to the distinct layers seen in CBABC_C and CBABC_A. Based on these observations, we infer that in the bridging conformation of ABC_A, chain pairs are oriented in opposite directions with a slight overlap between B and C blocks, as illustrated in Figure 9c. 

**Figure 10:**
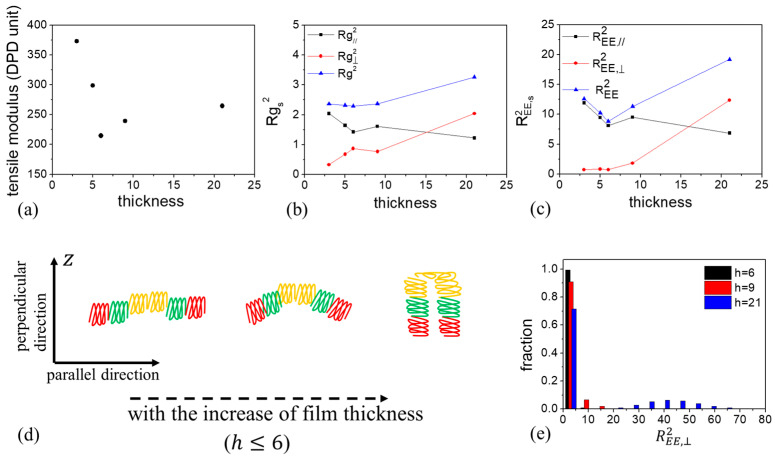
(**a**) Tensile modulus, (**b**) mean square radius of gyration $R_{g}^{2}$, and (**c**) mean square end-to-end distance $R_{EE}^{2}$ as a function of film thickness in the presence of an A block selective wall. (**d**) Variation in chain topology as film thickness increases from *h* = 3 to 6. (**e**) Histogram showing the perpendicular components $R_{EE,⊥}^{2}$ with various film thickness.

We further investigated the impact of film thickness on the tensile modulus in the presence of an A-selective wall (CBABC_A). Figure 10a displays the tensile modulus as a function of film thickness. The tensile modulus decreases for film thicknesses where h<6, then increases as *h* Increases. At h=6, the lamellae exhibit the lowest tensile modulus. Based on our previous analysis, we understand that the looping chain morphology predominates. As the film thickness changes, lamellae structures with varying periods and layer compositions are observed (as shown in Figure 5a and Figure 6). Does this structural variation influence chain conformations and, subsequently, the mechanical properties of the film? To address this question, we examined $R_{g}^{2}$ and $R_{EE}^{2}$ to analyze chain conformations.

When h<6, the mean square radius gyration remains nearly constant, while the two components of $R_{g}^{2}$ change inversely (Figure 10b). This implies that the chains elongate on the xy plane parallel to the wall for ultrathin films. The parallel alignment of chains results in a high tensile modulus. $R_{EE,⊥}^{2}$ remains almost unchanged with the film thickness (Figure 10c). However, the increase in $R_{g,⊥}^{2}$, coupled with the decrease in $R_{g,∥}^{2}$ and $R_{EE,∥}^{2}$ with h, indicates that the chains bend in the z direction, with both ends simultaneously approaching in the parallel direction (Figure 10d). This alteration in chain conformation leads to a reduction in tensile modulus. As film thickness increases from *h* = 6 to 21, the end-to-end distance of the chain gradually expands in the perpendicular (z) direction (Figure 10c). The probability distribution function reveals that the number of chains for which $R_{EE,⊥}^{2}$ < 5 decreases with film thickness, suggesting that more chains adopt a bridge conformation as h increases. The tensile modulus was found to rise with the ratio of chains exhibiting bridging conformation. Our findings align with the hypothesis proposed by Masten et al. [35], which suggests that the variations in mechanical properties can be attributed to the ratio between bridging and looping chains in the polymer conformation. 

In summary, the mechanical properties of the film are closely related to chain conformation. At very thin film thicknesses, polymer chains align parallel in the xy plane, resulting in a high tensile modulus. As the film thickness increases, polymer chains begin to fold in the z direction, adopting a loop conformation, which causes a decrease in the tensile modulus. With further increases in thickness, some chains start to extend in the z direction. The proportion of chains in a bridging conformation relative to those in a looping conformation grows, leading to a rise in the tensile modulus.

#### 3.2.2. B-Block-Selective Wall

**Figure 11:**
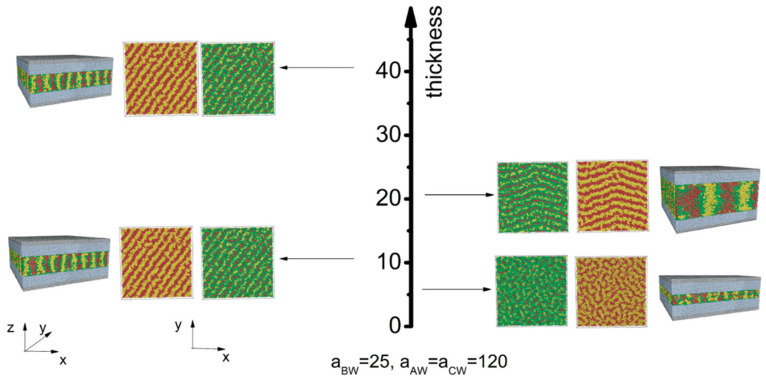
Thickness-dependent self-assembly morphology of CBABC pentablock terpolymers in the presence of a B-selective wall. The corresponding film thicknesses are h=6, h=11, h=21, and h=41.

**Figure 12:**
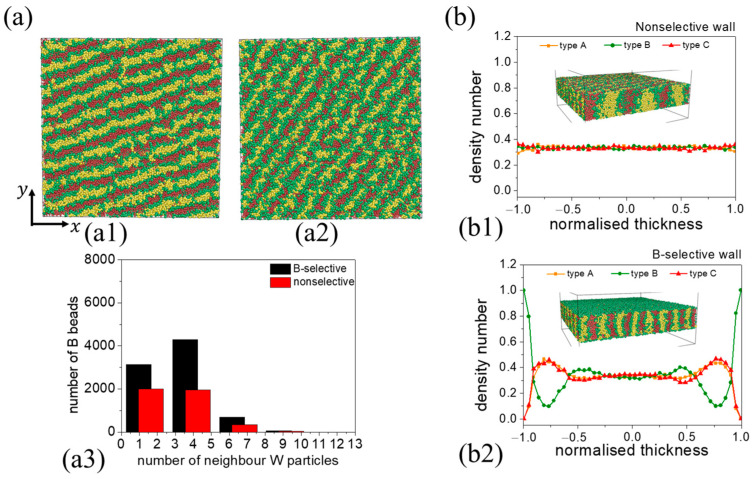
(**a**) Self-assembly morphology obtained in the case of a (**a1**) nonselective wall and (**a2**) B-selective wall with a film thickness of h=11. (**a3**) A histogram showing the number of neighboring wall particles around B beads. (**b**) Density number profile for lamella structure in the z direction in the case of a (**b1**) nonselective wall and (**b2**) B-selective wall.

We further explored the self-assembly behavior when encountering a B-selective wall. Figure 11 presents the morphological transition in relation to film thickness. Due to the wall’s preference for B blocks, a layer of B beads consistently forms at the interface between the polymer and the wall, regardless of film thickness. At h=6, a disordered phase emerges. When h>6, polymer chains consistently form a perpendicular lamellae structure across all film thicknesses. This behavior can be attributed to the combined effects of chain topology and wall preference. Within the polymer chain, the B block is flanked by the A and C blocks. Given that the wall is B-block-selective, most B beads tend to contact the wall to reduce interaction enthalpy. Consequently, the formation of a perpendicular lamellae structure emerges as the most energetically favorable arrangement. This lamellae structure bears resemblance to that formed with a nonselective wall (Figure 12(a1,a2)).

To distinguish between these two kinds of perpendicular lamellae structures, we compared their structural morphology, wall affinity, and density number at a consistent film thickness of h=11. The wall’s affinity for B blocks can be assessed by counting the number of neighboring W beads (wall beads) surrounding each B bead. In this context, a “neighbor” is defined as any bead located within the cut-off radius $r_{c}$. The distribution of B beads based on the count of neighboring W beads for the two scenarios is depicted in Figure 12(a3). In the B-selective case, there are more B–W interaction pairs, with a majority of B beads having between three and five neighboring wall particles, indicating a close association of B beads with the wall. Further insights can be gained from the number density distribution profile in the z direction in both cases. As illustrated in Figure 12(b1), when the wall is nonselective, there is a uniform distribution of all bead types in the z direction. However, in the case where the wall has a preference for B blocks, a distinct layer of B beads can be observed at the wall–polymer interface. Adjacent to the B peaks, a minor peak consisting of A and C blocks can also be observed (Figure 12(b2)).

We further observed the evolution process of perpendicular lamellae obtained in the case of B-selective and nonselective walls. With the B-selective wall, two layers of B beads form quickly next to the wall, while A beads and C beads aggregate into small clusters at t=10,000 timesteps (Figure S6(b1,b2)). These small clusters gradually merge to form larger aggregates (Figure S6(c1–c3,d1–d3)). The morphology gradually evolves and finally forms a perpendicular lamellar structure (Figure S6(e1–e3)). A similar evolution process was observed for the nonselective wall. The chains first aggregate into small clusters with distinct A, B, and C regions. These small clusters gradually merge and form lamellae structures (Figure S7). Unlike the B-selective wall, where individual B layers are formed next to the wall, the nonselective wall, being neutral to all blocks, does not lead to the formation of separate B layers next to the wall.

## 4. Conclusions

In this study, we utilized the dissipative particle dynamics (DPD) method to investigate the influence of film thickness on the equilibrium structures of $C_{3}B_{3}A_{6}B_{3}C_{3}$ pentablock terpolymers under 1D confinement. Two scenarios based on wall properties have been discussed.

For nonselective walls, the dominant configuration is one where lamellae are oriented perpendicular to the wall. Depending on the film thickness, block immiscibility, and polymer–wall interaction strength, various structures can be observed, including perpendicular lamellae, fingerprint lamellae, and cross-packed lamellae. Notably, the order–disorder transition (ODT) temperature is influenced by the interaction between the polymer and the wall in thin confinement scenarios.

For selective walls, we identified two distinct lamellar structures. When the wall preferred A or C blocks, parallel lamellae with v periods were observed, oriented parallel to the wall. The tensile properties of these lamellae are closely related to chain conformation, with a higher proportion of looping chains exhibiting improved elasticity. Conversely, when the wall had an affinity for B blocks, a perpendicular lamellar structure was observed, characterized by a single B layer aligned parallel to the wall.

In our current work, our focus has been on cases with two identical preferential surfaces confined to the top and bottom. In experiments, when forming a film, the two confined surfaces may be different and may have distinct preferences for the polymers. For example, one surface could be a substrate, and the other could be air. This means their interactions with the polymers will be different. Therefore, in our future work, we will explore the self-assembly behavior of polymers in the presence of surfaces with different types of selectivity.

In summary, the orientation of lamellae structures can be manipulated by altering wall properties or block selectivity. To fine-tune the mechanical properties of these structures, two methods are available: (1) adjusting the wall selectivity towards either the two free end blocks or the center blocks; (2) modifying the film thickness. These adjustments allow polymer chains to exhibit varying ratios of bridging-to-looping chains.

## Figures and Tables

**Table 1:** Interaction parameters *a_ij_*.

*a_ij_* (DPD Units)	A	B	C	W (Wall)
A	25			
B	40/80	25		
C	40/80	40/80	25	
W (wall)	25/120	25/120	25/120	25

**Table 2:** Mean square radius of gyration $R_{g}^{2}$ for polymers at different types of wall selectivity, $R_{g,∥}^{2}$ and $R_{g,⊥}^{2}$ are two components of $R_{g}^{2}$ parallel and perpendicular to the wall.

	$R_{g,s}^{2}$	$R_{g,∥}^{2}$	$R_{g,⊥}^{2}$	$R_{g}^{2}$
Chain				
ABC_A		1.64	1.02	2.66
CBABC_C		1.37	1.84	3.21
CBABC_A		1.42	0.87	2.29

**Table 3:** Mean square end-to-end distance $R_{EE}^{2}$ for polymers at different types of wall selectivity. $R_{EE,∥}^{2}$ and $R_{EE,⊥}^{2}$ are two components of $R_{EE}^{2}$ parallel and perpendicular to the wall.

	$R_{EE,s}^{2}$	$R_{EE,∥}^{2}$	$R_{EE,⊥}^{2}$	$R_{EE}^{2}$
Chain				
ABC_A		9.67	6.43	16.09
CBABC_C		7.91	12.77	20.68
CBABC_A		8.10	0.73	8.83

## Data Availability

The data presented in this study are available on request from the corresponding author. The data are not publicly available due to privacy.

## Supplementary Materials

The following supporting information can be downloaded at: https://www.mdpi.com/article/10.3390/ma16216862/s1, Figure S1: Density number distribution of self-assembly morphology of CBABC pentablock terpolymer along the z axis at different film thicknesses. The normalized thickness is defined as $\frac{z-0.5*h}{0.5*h}$, where z is the distance to the upper wall and *h* is the film thickness. (a) A-selective wall, $a_{AW}=25$; (b) C-selective wall, $a_{CW}=25$; Figure S2: Self-assembly morphologies and the corresponding density number distribution along the *z* axis at ultrathin film thicknesses in the case of a C-selective wall: (a) h = 5; (b) h = 4; (c) h = 3. The normalized thickness is defined as $\frac{z-0.5*h}{0.5*h}$, where *z* is the distance to the upper wall and h is the film thickness; Figure S3. Evolution of the parallel lamellar morphology formed at a film thickness of h=6 in the case of an A-selective wall; Figure S4. Evolution of the parallel lamellar morphology formed at a film thickness of h=21 in the case of an A-selective wall; Figure S5. Schematic showing the different types of chain conformation: (a) bridging conformation and (b) looping conformation; Figure S6. Evolution of the perpendicular lamellar morphology formed at a film thickness of h=11 in the case of a B-selective wall.; Figure S7. Evolution of the perpendicular lamellar morphology formed at a film thickness of h=11 in the case of a non-selective wall.
