# Supplementary material for: Effect of Film Thickness on the Self-Assembly of CBABC Symmetric Pentablock Terpolymer Melts under 1D Confinement: A Dissipative Particle Dynamic Study
Source: Materials (Basel). 2023 Oct 25;16(21):6862. doi: 10.3390/ma16216862 (PMC10648495; doi:10.3390/ma16216862)
Supplement: Supplementary file 1 [file materials-16-06862-s001.zip › materials-2674425-supplementary.pdf]

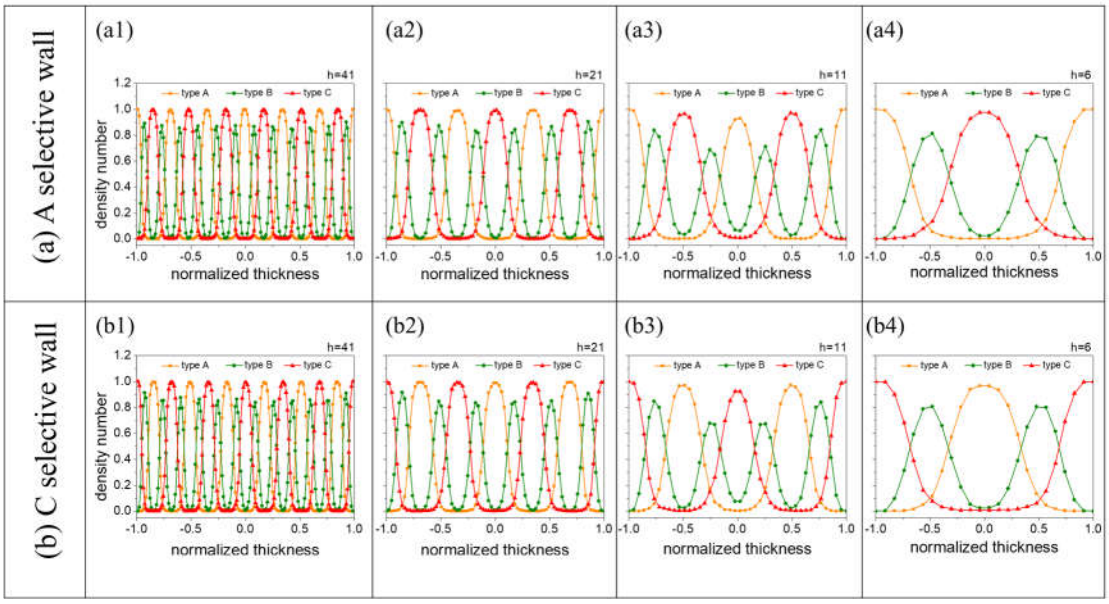

Figure S1. Density number distribution of self-assembly morphology of CBABC pentablock terpolymer along the z axis at different film thickness. The normalized thickness is defined as $\frac{z-0.5*h}{0.5*h}$, where z is the distance to the upper wall and h is the film thickness. (a) A-selective wall $a_{AW} = 25$; (b) C-selective wall $a_{CW} = 25$.

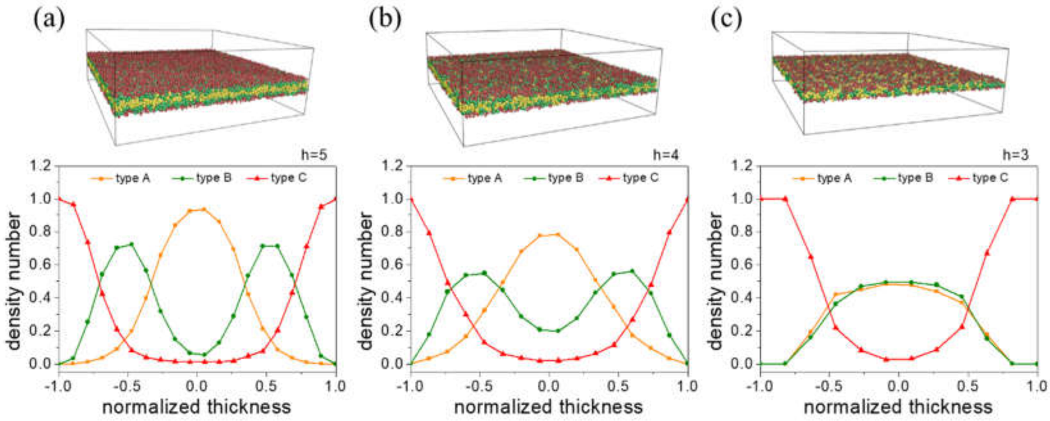

Figure S2. Self-assembly morphologies and the corresponding density number distribution along z axis at ultrathin film thickness (a) h=5; (b) h=4; (c) h=3 under C-selective wall. The normalized thickness is defined as $\frac{z-0.5*h}{0.5*h}$, where z is the distance to the upper wall and h is the film thickness.

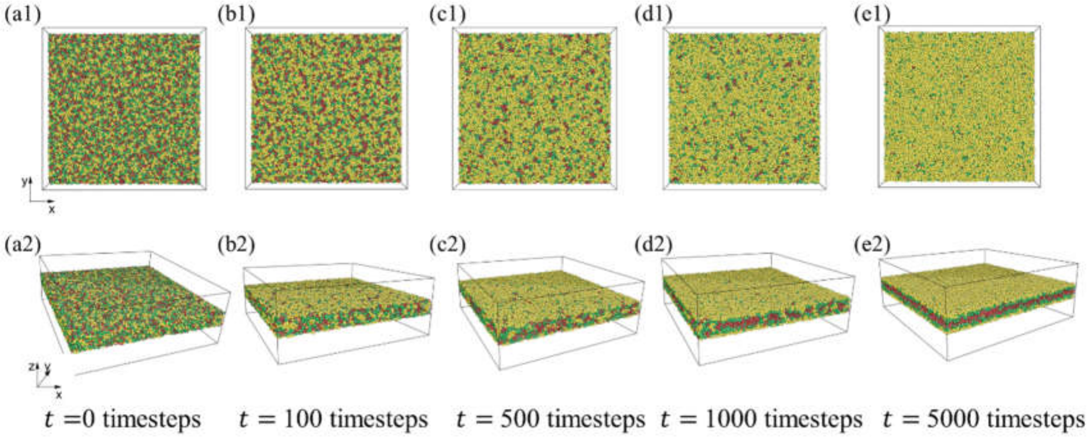

Figure S3. Evolution of the parallel lamellar morphology formed at film thickness h = 6 under A-selective wall.

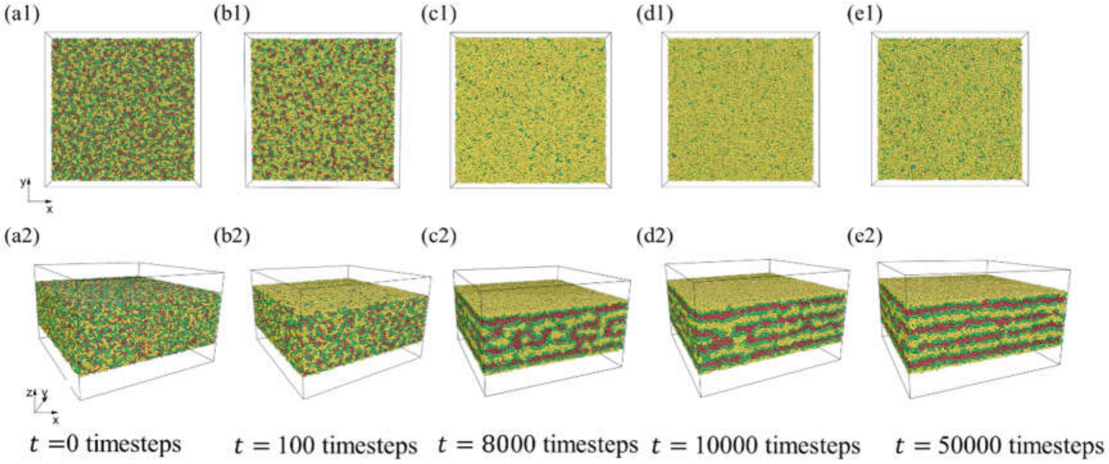

Figure S4. Evolution of the parallel lamellar morphology formed at film thickness h = 21 under A-selective wall.

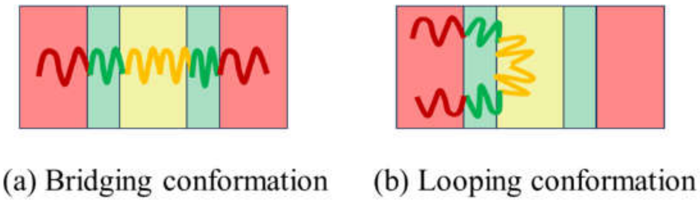

Figure S5. Schematic showing the different types of chain conformation: (a) bridging conformation and (b) looping conformation.

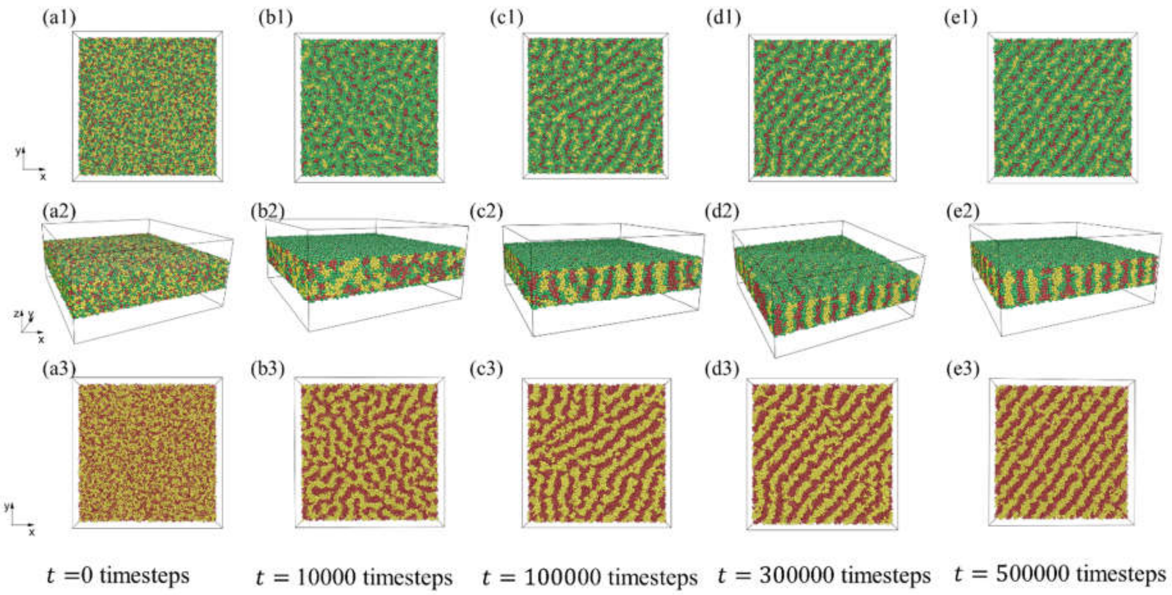

Figure S6. Evolution of the perpendicular lamellar morphology formed at film thickness $h = 11$ under B-selective wall.

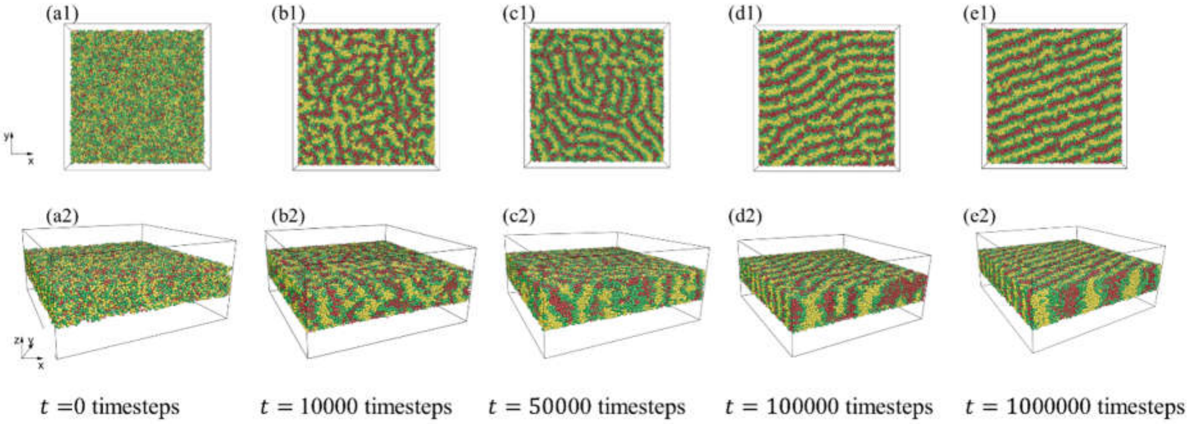

Figure S7. Evolution of the perpendicular lamellar morphology formed at film thickness $h = 11$ under non-selective wall.
